# Lasting Impact of an Ephemeral Organ: The Role of the Placenta in Fetal Programming

**DOI:** 10.1289/ehp.124-A124

**Published:** 2016-07-01

**Authors:** Lindsey Konkel

**Affiliations:** Lindsey Konkel is a New Jersey–based journalist who reports on science, health, and the environment.

During the winter of 1944–1945 a brutal cold snap and the World War II German occupation curtailed food shipments across the Netherlands. The Dutch plunged into severe famine, with adult food rations dwindling to just 400 calories a day in some areas. Babies conceived during the Dutch “Hunger Winter” were born shorter, thinner, and with smaller heads and placentas than babies born before or conceived afterward. Years later, the famine babies were more likely to suffer from obesity, diabetes, and heart disease than peers born in the years shortly after the famine. They tended to die younger.^1^

**Figure:**
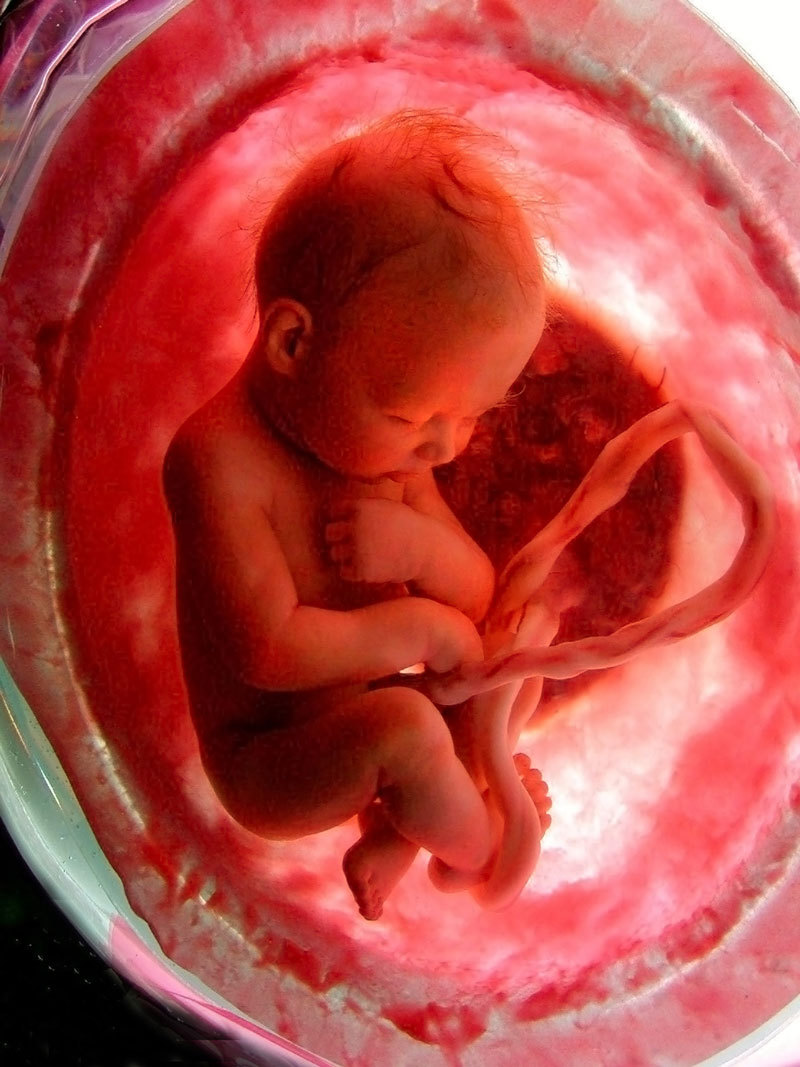
Recent advances in molecular and imaging technologies, “omics” fields, and data sciences are offering researchers an unprecedented look at the placenta, the master regulator of the fetal environment. © EPA/National Geographic Channel/Alamy

The Dutch famine provided early clues that environmental stressors encountered in the womb could determine disease risk in adulthood—a phenomenon known as fetal programming. Over the next several decades, evidence grew that a number of chronic conditions, including asthma, cancer, and neurodevelopmental disorders, might be traced back to environmental exposures in the womb.^2^ Experts have called this emerging paradigm “developmental origins of health and disease,” or DOHaD.^2^ ^,^ ^3^

Fetal programming is one of the most rapidly expanding areas of biomedical research.^4^ Yet the mechanisms underlying this phenomenon have remained murky. Epigenetic alterations—changes that affect how genes are expressed but not the DNA itself—may underlie many of these processes. Researchers are now exploring DOHaD in relation to the structure, function, and epigenome of an often overlooked but essential organ—the placenta.

Since early times humans have surmised an important, if not mystical, role for the ephemeral organ that connects the fetus, via the umbilical cord, to the mother’s blood supply and provides for the passage of nutrients and oxygen to the fetus from the mother. Ancient Egyptians revered the placenta as the “External Soul,” while Hebrew Scriptures called it the “Bundle of Life.” The ancient Greeks settled on a more physical description for the glistening crimson sac: They named it the “placenta,” or “flat cake.”^5^

Yet for millennia, the placenta remained one of the least understood human organs. Gross placental abnormalities were known to have immediate health consequences for mother and fetus, yet no one suspected that even a seemingly normal placenta could influence the lifelong health of the child beyond the prenatal period. “Until very recently, the placenta was thought of by the scientific community as this static plug connecting the fetus to the maternal circulation,” says Graham Burton, a placentologist at the University of Cambridge, United Kingdom. That view is changing.

Recent advances in molecular and imaging technologies, “omics” fields, and data sciences are offering researchers an unprecedented look at the placenta as a dynamic organ whose molecular structure and function change throughout pregnancy.^6^ Scientists now know that the placenta mediates fetal interactions with the maternal immune system and exposures to compounds in the mother’s blood, in addition to its role in nutrient and waste transfer between the mother and fetus.^7^ It also functions as a neuroendocrine organ that produces hormones and other important molecules to spur fetal growth and development.^7^ The placenta, in essence, may be the master regulator of the fetal environment.^8^

## A New View of Prenatal Exposures

The thalidomide crisis of the mid-twentieth century established the vulnerability of the fetus during the prenatal period and provided evidence that the placenta was not an impervious barrier against toxic exposures. Doctors prescribed thalidomide to pregnant women for morning sickness for four years before determining in 1961 that it caused many of the women’s babies to be born without arms or legs.^9^

Researchers soon found that low levels of chemicals with more subtle effects than thalidomide—for instance, lead, mercury, polychlorinated biphenyls (PCBs), and nicotine—also could cross the placenta and enter the fetal blood supply, causing damage.^10^ Once inside the placenta, researchers speculated that these chemicals had direct access to the fetal brain and other developing tissues.

Even still, says Thad Schug, a health scientist administrator for the National Institute of Environmental Health Sciences, “we assumed for a long time that things either did or didn’t cross the placenta. They either did or didn’t enter the developing fetus.” No one was really paying attention to how the placenta responded to those contaminants and whether they might alter the function of the placenta itself.

The dominant model of fetal toxicology assumed that chemicals passed through the placenta to reach the fetus but did not change placental function. Yet this model could not totally explain the developmental effects and associations that researchers were finding. Namely, experimental and observational studies pointed toward vulnerable periods for brain development and sexual differentiation in the earliest weeks of pregnancy,^11^ but studies of placental structure suggested that toxicants probably did not pass through the placenta in these early stages.^1^ In the past decade, the static fetal model has begun to change as technological advances have allowed researchers to get a better look at the placenta’s structure and molecular profile.

The placenta undergoes a number of changes during pregnancy. In its earliest days, it looks and functions very differently from the organ seen at birth. One of the placenta’s best-established roles—as a conduit of fetal nutrition—does not start until several weeks into pregnancy. During the first weeks, the developing embryo receives nourishment from the glands lining the uterus while the placental cells implant into the uterine wall and lay a sturdy framework for the maternal–fetal interface. During this early period of development, when the major organs are differentiating, the embryo has very limited access to the mother’s circulation. Only at about 10 weeks after conception do the placental cells connect to the maternal blood supply. At that point, the placental cells, bathed in maternal blood, begin to transfer oxygen, nutrients, and other molecules to the fetus.^1^ Unfortunately, this newly formed connection also allows for the passage of environmental chemicals from maternal blood to the fetus.

So how might chemical exposures influence development during this critical period without directly contacting the embryo? From the start of pregnancy, the placenta is a tiny factory, making and secreting chemicals such as human chorionic gonadotropin (hCG).^12^ hCG is an important placental signaling hormone that plays a variety of roles during pregnancy, the best known of which is to help maintain the production of steroid hormones by the mother’s ovaries; this sustains early pregnancy. hCG is also important in stimulating the production of testosterone, which helps direct sexual differentiation in male fetuses. Jennifer Adibi, a molecular biologist and epidemiologist at the University of Pittsburgh, suspected that endocrine-active chemicals such as *ortho*-phthalates, which are known to alter sexual differentiation in rodents,^13^ ^,^ ^14^ ^,^ ^15^ ^,^ ^16^ might do so by altering hCG production by the placenta.

To examine this hypothesis, Adibi dosed placental cells in culture with levels of phthalates comparable to those circulating in the bodies of most Americans and found that exposed cells produced less hCG than nonexposed cells. She then compared levels of phthalates in the blood of 541 pregnant women with their newborn babies’ anogenital distance (AGD; the distance between the anus and genitals). Women with higher blood phthalate levels tended to have higher levels of hCG in the first trimester. AGD is normally longer in male newborns than in females, but these women tended to have sons with a shorter-than-normal AGD (a sign of feminization) or daughters with a longer-than-normal AGD (a sign of masculinization).^17^ Variations in AGD have been associated with differences in some measures of reproductive function in both males and females, although the clinical impacts are unknown.^18^ ^,^ ^19^

**Figure:**
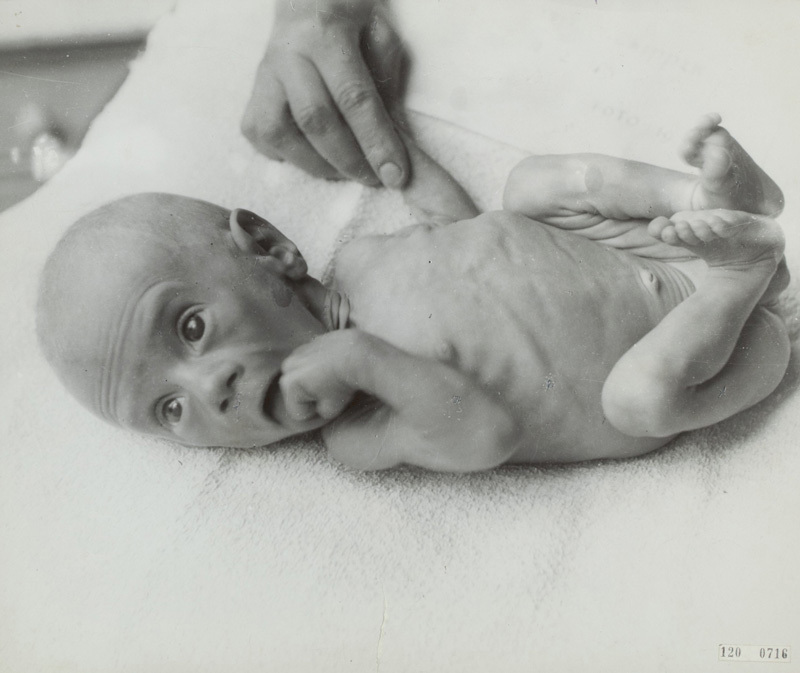
Studies of infants conceived during the Dutch “Hunger Winter” provided some of the earliest clues that prenatal stress could affect health much later in life. © Nationaal Archief

These new findings offer evidence that phthalates and possibly other endocrine-disrupting chemicals may be able to affect fetal development indirectly by altering placental function. Like direct effects, these indirect effects may influence disease development later in life.^17^ “We’re beginning to see that how the placenta deals with environmental change may be an important and previously overlooked component of fetal programming,” Adibi says.

## Markers and Mechanisms

In addition to the Dutch famine studies, observational research carried out in the second half of the twentieth century linked gross characteristics such as infant birth weight and placental size to adult health outcomes.^20^ Those studies formed the scientific basis for what later became the DOHaD hypothesis.

In the early 1990s British epidemiologist David Barker reported that men and women in a county in southern England who had been born with low birth weight were more likely to develop type 2 diabetes and high blood pressure and to die of heart disease in later life than their normal-birth-weight counterparts.^21^ Barker called this the “thrifty phenotype,” where the fetus faced with malnutrition develops certain metabolic traits that help it to adapt to conditions of continued food shortage. These same traits put them at a disadvantage later in life, in a world of plentiful food, where they tended to suffer from excessive weight gain and associated chronic diseases. He hypothesized that the quality of the intrauterine environment could permanently alter the physiology and metabolism of the fetus and thereby impact the lifelong health trajectory of the child.

The early work of Barker and others gave us the 10,000-foot view of DOHaD. Researchers are now getting a close-up look at the epigenetic mechanisms that are thought to underlie some of those early morphological observations. Epigenetic regulation of gene expression can be accomplished by direct methylation of DNA. In methylation, small molecules known as “methyl groups” attach to genes in a specific pattern. Methylation can turn genes on or off directly at the level of DNA and affect whether the DNA can transcribe messenger RNA (mRNA). It is the mRNA that then makes proteins that direct cellular structure and function.^7^

Once transcribed, mRNA can be “silenced” by microRNA (miRNA), tiny noncoding strands of RNA that help to regulate gene expression. miRNA does not alter the genetic code but rather affects its expression, providing another mechanism for epigenetic regulation.^7^

Different tissues in the body have different signature DNA methylation patterns. These methylation patterns form the basis of an individual’s epigenome.^8^ Human studies have shown that environmental exposures to endocrine-active chemicals, heavy metals, stress, and malnutrition may influence DNA methylation patterns in fetal umbilical cord blood.^8^ Researchers are now getting a handle on normal and aberrant patterns of DNA methylation in the placenta, the first complex organ to form during development.^7^ “The placenta represents a higher level of control of fetal programming and a more broad range of impacts than other tissues,” says Carmen Marsit, an environmental epigeneticist at Emory University.

Marsit looks at the relationship between *in utero* exposures to environmental contaminants, such as arsenic, and DNA methylation patterns. Finding where differences in methylation occur helps researchers to identify specific candidate genes that may play important roles in exposure pathways.^22^

Epigenetic studies are starting to show links between the placental epigenome and a number of infant health markers. Variations in DNA methylation patterns in certain gene regions have been associated with infant birth weight, gestational age at birth, and neurobehavioral measures.^7^ “The next important step,” says Marsit, “is to understand the functional implications of these epigenetic changes—for instance, whether changes to DNA methylation or miRNA expression patterns alter gene expression in ways that matter to health.”

Rebecca Fry, a toxicologist at the University of North Carolina at Chapel Hill, searches for molecular clues in compromised placentas to help her determine how prenatal exposures to toxic metals may influence disease susceptibility. She has found associations between preeclampsia and placental levels of metals including cadmium.^23^ Preeclampsia is a pregnancy complication that results in decreased oxygenation and metabolic stress for the fetus, hypertension for the mother, and later risk for heart disease and stroke in the child. This condition affects about 3–7% of pregnancies.^24^

Doctors do not know exactly what causes preeclampsia, although the condition has been associated with improper blood vessel formation in the placenta. The only way to stop preeclampsia is to deliver the placenta, so labor is often induced in preeclamptic women, even if this means their children are born prematurely. “These infants are set up for many health complications later on, including problems related to neurodevelopment,” Fry says.

**Figure:**
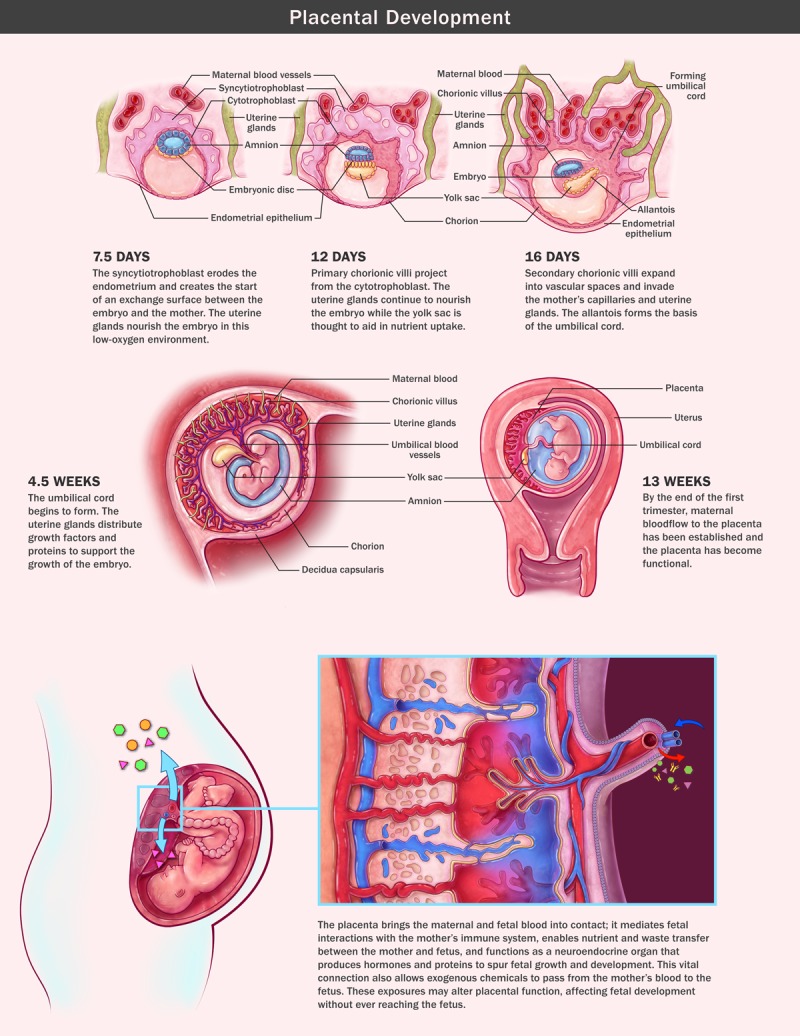
Placental Development

Fry’s studies assessing the functional consequences of epigenetic patterns in the placentas of mothers with preeclampsia may help to elucidate the biological pathways responsible for observed associations between toxic metals and health outcomes including infant growth, neurodevelopment, and immune function. “If we first understand which biological pathways are changed, we can begin to look for therapeutics to influence the faulty expression of those genes,” she says.^25^

**Figure:**
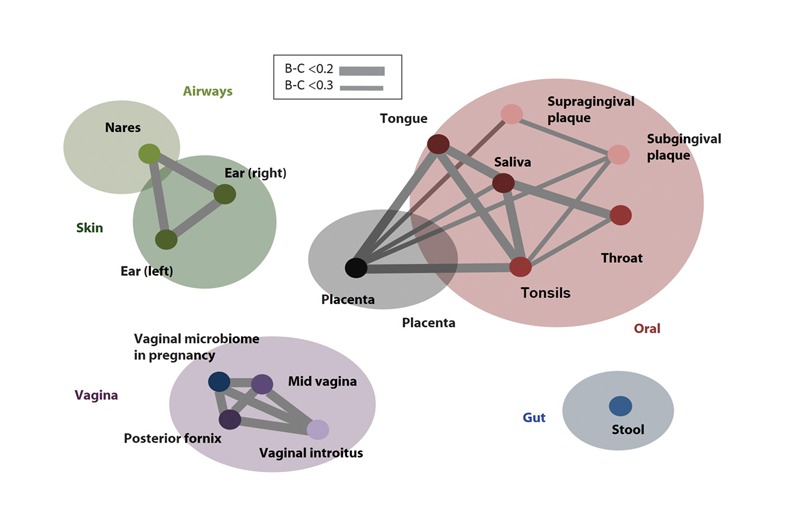
In one study, the placental microbiome had a similar taxonomic profile as the oral microbiome, illustrated here by Bray-Curtis (B-C) dissimilarity. B-C dissimilarity reflects the extent to which two sites have dissimilar compositions, with 0 indicating total similarity and 1 indicating total dissimilarity—the thicker the connecting line, the greater the similarity of the taxonomic profile. Source: Aagard et al. (2014)^38^

## A Unique Placental Microbiome?

Scientists have long thought the uterus was a sterile environment, but it turns out the placenta may not be germ-free. Recent studies suggest that all placentas contain a small amount of bacteria. In 2014 a team led by Kjersti Aagaard, a fetal medicine specialist at Baylor College of Medicine, found a distinct microbial signature among the placentas from 320 healthy human pregnancies.^38^ A study published earlier this year suggested that these placental microbes may begin to colonize the human gut shortly before birth.^39^

The findings are controversial. Some researchers caution that microbial discoveries in samples with few bacteria—such as the placenta—could be the result of DNA contamination.^40^,^41^ Questions remain about whether the bacteria in the human placenta even constitute a true microbiome—a persistent and distinct community of microbial residents—and how those bacteria got there.

Aagaard now is using laboratory animals to infer how environmental factors, including maternal diet, can impact this potential placental microbiome. Efforts to understand the placental microbiome may provide critical insights about DOHaD. “The microbiome helps form our metabolic premise,” Aagaard says. “If you disturb that metabolic milieu during a critical window of development, it’s going to have a lifelong impact.”

## Molecular Influences on Outcomes

Studies of where and how epigenetic modifications occur in the placenta are giving researchers a better understanding of the ephemeral organ’s molecular landscape and the functional relevance of these changes.

Recent studies have connected miRNA to several placenta-related conditions, including preeclampsia and fetal growth restriction. Cell studies have further shown that placental exposure to a number of environmental stressors, including metals and bisphenol A (BPA), may alter miRNA expression.^7^ In one study, altered miRNA expression in cells dosed with environmentally relevant concentrations of BPA caused the cells to become more sensitive to DNA-damaging molecules.^26^

Karin Michels, an epidemiologist at Harvard who studies early-life risk factors for breast cancer, says that miRNAs may provide very early markers of disease risk. “MicroRNAs could help us to define cancer mechanisms that are set *in utero*,” says Michels. She recently showed that prenatal exposures to two classes of endocrine-disrupting chemicals—phthalates and phenols—were associated with differences in miRNA expression patterns in the recently delivered placentas of nearly 200 women, suggesting potential mechanisms for toxicity in humans.^27^

Genomic imprinting could provide clues, too. For most genes, we inherit two working copies (or alleles)—one from each parent. With imprinted genes, only one of the inherited alleles is functional; the other is silenced by DNA methylation. The copy of the gene that is silenced depends on which parent the allele was inherited from. Genomic imprinting is one of the few known mechanisms for transgenerational epigenetic inheritance—the process by which epigenetic alterations can be passed from one generation to the next.

Many imprinted genes are involved in placental development and fetal growth.^28^ Problems with imprinted genes have been associated with a number of disorders, including diabetes, cancer, reproductive diseases, and behavioral disorders.^28^ In experimental studies, exposures to toxic metals and endocrine-active chemicals have been associated with differences in the regulation of imprinted genes in the placenta,^29^ but it’s not yet clear what these changes might mean for the long-term health of the growing fetus.

A consistent feature of the DOHaD hypothesis is the occurrence of sex-specific differences in the appearance and progression of many diseases.^30^ There are also sex-specific differences in neurodevelopment and the acquisition of cognitive skills. Experimental models suggest that exposure to environmental insults early in life can program sex-specific differences in adult heart disease risk.^31^ Yet, it’s unclear exactly how biological sex influences the mechanisms underlying disease progression.^32^ Researchers believe some of these differences can be traced to the placenta.

Many mammals, including humans, show sex-specific differences in placental structure and function. Cheryl Rosenfeld, a veterinarian and environmental scientist at the University of Missouri, explains that there may be differences in the way that placentas for male versus female fetuses deal with environmental stresses, differences she says could set boys and girls on different health trajectories.

Rosenfeld showed that placentas supporting female mouse pups were more sensitive to changes in the maternal diet than placentas of male pups.^33^ Further studies on maternal diet in mice found differences in DNA methylation and gene expression patterns in the placentas of male and female offspring, and associated sex-dependent differences in how offspring responded to a high-fat diet.^34^ Maternal stress in rodents likewise led to different patterns of epigenetic regulation and gene expression in male and female placentas, with male offspring going on to develop maladaptive inflammatory and behavioral responses to stress as adults.^35^

Researchers have hypothesized sex-specific differences in the way the placenta influences cardiovascular and brain development in humans, and the Dutch famine of 1944 provided evidence of a placental–heart relationship in humans. Men appear to be disproportionately affected by some cardiovascular and neurological disorders with suspected placental origins.^30^ Sons that were in the womb during peak famine months had oddly shaped placentas, and these changes correlated with the development of hypertension later in life—an association that was not found in daughters.^30^ However, few studies have investigated sex-dependent differences in placental responses to specific exposures.^30^

## Challenges and Opportunities

“All placentas serve as a buffer between the mom and the fetus, but no other organ has evolved to be so structurally different between different species,” Rosenfeld says. Molecular pathways that perform important functions in some species may not exist in others. For instance, mice do not produce hCG but have other ways of regulating functions directed by hCG in humans.

The diversity in placental structure makes it difficult for researchers to establish appropriate animal models to validate causal mechanisms. Placental diseases such as preeclampsia also can be hard to model in animals.

Researchers are looking to embryonic stem cells for potential experimental models. Human embryonic stem cells can be converted to placental cells, and these cells can be exposed to different stressors to replicate some of the conditions of human pregnancy. An *in vitro* approach can provide insight into disruptions taking place during the earliest stages of placental formation, before the organ is even attached to the uterus, Rosenfeld says.

Accessing the placenta during critical periods of fetal development presents another challenge. Most human placental studies are conducted on placentas that have been delivered at term, but a term placenta looks and functions very differently than a first-trimester placenta. “It’s like studying a heart after it has already stopped beating,” says David Weinberg, a translational scientist at the Eunice Kennedy Shriver National Institute of Child Health and Human Development. Weinberg says, “To really understand placental function and development, we need to be able to monitor it all across pregnancy.”

Weinberg is the project lead for the institute’s Human Placenta Project (HPP). The HPP was launched in 2014 to better understand how the placenta influences the health of the mother and fetus, both during pregnancy and beyond.^6^ HPP researchers are working to devise new technologies and methods that will enable noninvasive, real-time assessment of placental development and function across pregnancy. Many of the tools that are being developed will allow researchers to better evaluate environmental impacts on the placenta, Weinberg says.

One exciting approach, according to Weinberg, is the study of placenta-derived exosomes^36^—small cellular structures in biological fluids that relay messages from the placenta back to the mother. Researchers are looking at how these exosomes change across pregnancy by studying their abundance and structure. Such molecules may provide real-time markers of fetal health that can be measured in the mother’s blood.^37^

Beyond a better understanding of fetal programming, the placenta may have much to offer the broader research community, Weinberg says. Learning how placental cells proliferate and invade the wall of the uterus, for instance, could aid cancer researchers. “We believe that understanding those processes will not only benefit moms and fetuses but can lead to advances that extend far beyond pregnancy,” he says.
